# Revision of the Oriental species of *Calleida* Latreille (*sensu lato*). Part 2: the *C.discoidalis* species group (Coleoptera, Carabidae, Lebiini)

**DOI:** 10.3897/zookeys.806.30051

**Published:** 2018-12-13

**Authors:** Hongliang Shi, Achille Casale

**Affiliations:** 1 c/o Beijing Forestry University, College of Forestry, Beijing 100083, China Beijing Forestry University Beijing China; 2 Università di Sassari, Italy (Zoologia); private: Corso Raffaello 12, 10126 Torino, Italy Università di Sassari Sassari Italy

**Keywords:** *
Calleida
*, character evolution, Oriental, spermatheca, taxonomy

## Abstract

The *C.discoidalis* species group of the genus *Calleida* Latreille from Asia (in the sense of Casale and Shi 2018) is revised with six species recognized. Four new species are described: *C.piligera* Shi & Casale, **sp. n.** (type locality: Taiwan: Siling, 24.65°N, 121.42°E); *C.cochinchinae* Casale & Shi, **sp. n.** (type locality: Vietnam: “Cochinchina”); *C.yunnanensis* Shi & Casale, **sp. n.** (type locality: Yunnan: Caiyanghe, 22.60°N, 101.12°E); and *C.luzonensis* Casale & Shi, **sp. n.** (type locality: Philippines: Nagtipunan, 16.22°N, 121.60°E). *C.fukiensis* Jedlička, 1963 is confirmed as an available and valid species name, and C. *suensoni* Kirschenhofer, 1986 is newly synonymized with it. A phylogenetic analysis of Oriental *Calleida* species, based on adult morphological characters, is performed. The results show that the monophyly of most species groups in Oriental *Calleida* is accepted, but the *C.discoidalis* group appears polyphyletic and comprises three lineages. However, because many species relationships in the cladogram lack significant supporting, presently the *C.discoidalis* group was remained to use for morphological convenience. Five types of female reproductive tracts were recognized, corresponding to five branches in the cladogram.

## Introduction

*Calleida* Latreille, 1824 is a genus of LebiiniCalleidina (Coleoptera, Carabidae) with rich species diversity mainly distributed in tropical and subtropical regions of the Americas, Sub-Saharan Africa and Southeast Asia (Casale 1998). These small to medium-sized and beautiful carabid beetles generally have vivid metallic color, but species identifications are very difficult in many cases. Our first contribution to the Oriental species of *Calleida* (Casale & Shi, 2018) provided a primary infrageneric taxonomy with nine species groups recognized and species revisions of six species groups.

As the second part of our contributions to the Oriental species of *Calleida*, the present paper is mainly dedicated to revision of the *C.discoidalis* species group. This group is defined as follows: abdominal sternite VII with four or more setae in males (two or more on each side), six or more setae in females (three or more on each side); abdominal sternite VII notched in males (Figs 2, 5); endophallus with two short copulatory pieces. This species group contains six known species distributed in China, Laos, Vietnam, and the Philippines. Two of them were described previously, and four are here described as new species.

At the beginning of our work, we included all Oriental species with multisetose abdominal sternite VII (four or more setae on abdominal sternite VII in males and six or more in females) in the *C.discoidalis* group. But *C.puncticollis* Shi & Casale, although with multisetose abdominal sternite VII, was recognized as very different from all other members both from external and genital characters. Thus, we erected a separate group to accommodate this species (Casale and Shi 2018). Even so, the members of the so-called *C.discoidalis* group are easily distinguishable by external features and different to each other in female genital characters, but this group is only supported by one external morphological character. Based on this, the *C.discoidalis* group is inferred to be paraphyletic or polyphyletic, and seems to be composed of three lineages with different female genital characters. However, it is difficult to define these lineages as distinct groups or move them to other groups based on the external and male genital characters. Therefore, we proposed the *C.discoidalis* group as a simple “group of convenience” to make identifications of species and species groups easier (Casale and Shi 2018).

When studying morphologies of each species, we found that the female reproductive tract (especially the spermatheca) has very important value of inferring species relationships of the Oriental *Calleida*. Thus, a preliminary phylogenetic analysis was performed to solve the systematic position of each species of the non-monophyletic *C.discoidalis* group, and moreover to interpret female genitalic evolution and evaluate its taxonomic value in the genus.

## Materials and methods

### Materials

This contribution was based primarily on the examination of *Calleida* specimens from different collections which are indicated with abbreviations:

**BMNH** British Museum of Natural History, London, U.K.

**CCA** Collection of Achille Casale, Torino, Italy

**CCCC** Collection of Changchin Chen, Tianjin, China

**CDG** Collection of Augusto Degiovanni, Bubano di Mordano (Modena), Italy

**CPB** Collection of Peter Bulirsch, Prague, Czech Republic

**CRS** Collection of Riccardo Sciaky, Milano, Italy

**HBUM** Museum of Hebei University, Baoding, China

**IZAS** Institute of Zoology, Chinese Academy of Science, Beijing, China

**MNHN** Muséum National d’Histoire Naturelle, Paris, France

**NHMW** Naturhistorisches Museum, Wien, Austria

**NMNST** National Museum of Natural Science, Taibei, China

**NMPC** Národní Přírodovědecké Muzeum, Prague, Czech Republic

**SCAU** South China Agriculture University, Guangzhou, China

**SMTD** Staatliches Museum für Tierkunde

**SYUM** Sun Yat-sen University Museum, Guangzhou, China

**ZMFK** Zoologische Forschungsmuseum Alexander Koenig

**ZSM** Zoologische Staatssammlungen, München, Germany

### Methods

The methods, terminology and taxonomic treatment follow our previous work (Casale and Shi 2018). The phylogenetic reconstruction was performed using WIN-PAUP v. 4.0b10 software. The cladograms were created with FigTree v. 1.4.0 and trees were edited with Adobe Photoshop software. Parameters used in the analyses are listed in associated text.

### Abbreviations

**TL**: body total length, from the anterior margin of clypeus to the apex of elytra, measured along the suture. **L**: overall length, from apex of mandibles to apex of elytra, measured along the suture. **PL**: length of pronotum, as linear distance from the anterior to the basal margin, measured along the midline. **PW**: maximum width of pronotum, as greatest transverse distance of pronotum. **EL**: length of elytra, as linear distance from the basal ridge to the apex, measured along the suture. **EW**: maximum width of elytra, as greatest transverse distance of two closed elytra.

## Taxonomy

### Key to species of *Calleidadiscoidalis* species group (in the sense of Casale and Shi 2018)

**Table:** 

1	All intervals of elytra with strong metallic lustre, uniformly green, bluish or cupreous, without any trace of a dull reddish patch on disc	**2**
–	Elytra metallic green or bluish, with a reddish patch on the posterior half of disc, sometimes the reddish patch vague or narrow, but at least the inner three intervals with metallic lustre very faint (Figs 22, 23)	**4**
2	Elytra with basal ridge incomplete, extended only from shoulder to the fourth interval; elytral disc metallic bluish green to bluish purple; basal three antennomeres reddish yellow, the rest ones distinctly darkened; femora distinctly bicolor, with basal part yellowish and apical part almost black (Fig. 2); abdominal sternite VII with six or rarely seven setae in females (Casale and Shi 2018: fig. 5). S. China	[**1] *Calleidafukiensis* Jedlička**
–	Elytra with basal ridge complete, extended from shoulder to the parascutellar stria; elytral disc metallic green to cupreous; antennae and femora uniformly reddish, or with apical antennomeres and apex of femora only weakly darkened; abdominal sternite VII with eight or more setae in females (Fig. 13)	2
3	Head and pronotum dark brown, without trace of metallic lustre (Figs 9–11); elytra metallic green, or with cupreous reflection; body form stouter (EL/EW = 1.65–1.75). S. China (Taiwan, Shaanxi, Shanghai, Guangxi, Guizhou, Guangdong, Sichuan)	[**2] *Calleidapiligera* Shi & Casale, sp. n.**
–	Dorsal side, including head and pronotum, evenly metallic green (Fig. 19), with marked cupreous reflection, more evident at apex of elytra; body form slenderer (EL/EW = 1.84). S. Vietnam (“Cochinchina”)	[**3] *Calleidacochinchinae* Casale & Shi, sp. n.**
4	Head, pronotum and legs brownish, markedly darkened; elytra dark metallic green, with obvious reflections of cupreous red or purple on lateral and apical areas (Figs 22, 23). Pronotum elongate and narrow (PW/PL: 1.07–1.13), elytra very elongate (EL/EW = 1.72–1.75). China (Yunnan), Laos	[**4] *Calleidayunnanensis* Shi & Casale, sp. n.**
–	Head, pronotum, and legs red to reddish yellow; elytra bright metallic green or a little bluish, not reddish or cupreous on lateral or apical areas. Pronotum more transverse (ratio WP/PL = 1.17–1.24), elytra short and wider (EL/EW = 1.52–1.65). Philippine Islands	**5**
5	Head, pronotum and elytral patch dark reddish; elytral disc metallic bluish green; elytral patch usually wider, occupying the inner five or six intervals (Figs 32, 33); smaller beetles (L = 10 mm). Mindanao	[**5] *Calleidadiscoidalis*** Heller
–	Head, pronotum and elytral patch reddish yellow; elytral disc bright metallic green; elytral patch usually narrower, occupying the inner three to five intervals (Figs 36, 38, 39); larger beetles (L - 10.5–11.0). E. Luzon	[**6] *Calleidaluzonensis* Casale & Shi, sp. n.**

#### [1] Calleida fukiensis

Taxon classificationAnimaliaColeopteraCarabidae

Jedlička, 1963

Figs 1–8 , Map 1

Callida onoha ab. fukiensis Jedlička, 1953: 146 (type locality: China: “Fukien”; holotype deposited in ZFMK), unavailable name.
Callida fukiensis Jedlička, 1963: 437, available name.
Calleida suensoni Kirschenhofer, 1986: 324 (type locality: China: Zhejiang; holotype deposited in ZMUC, paratypes in NHMW), **new synonymy**.

##### Type materials.

**Figures 1–8.**
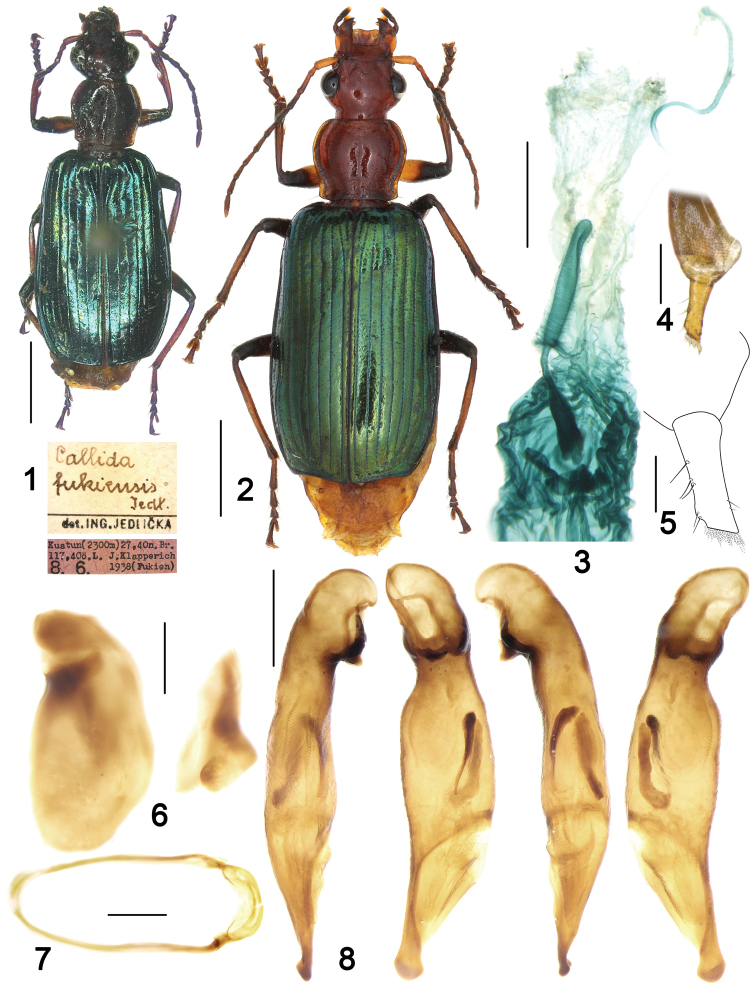
*Calleidafukiensis* Jedlička. **1** Habitus and labels of holotype (female, Fukien, ZMFK) **2** Habitus (female, Guizhou, IZAS) **3** Female reproductive tract (Shaanxi) **4** Gonocoxa (Shaanxi) **5** Gonocoxite II (Shaanxi) **6** Left and right parameres of aedeagus (Fujian) **7** male sternite IX (Fujian) **8** Median lobe of aedeagus, right-lateral, ventral, left-lateral, and dorsal views (Fujian). Scale bars: 2.0 mm (**1, 2**), 0.5 mm (**3, 7, 8**), 0.2 mm (**4, 6**), 0.1 mm (**5**).

***C.fukiensis*, holotype**: female, examined through photo (Fig. 1), “Kuatun (2300 m), 27,40n. Br. 117,40ö.L. J. Klapperich 8.6.1938 (Fukien)”, “Callidafukiensis Jedl., det. ING. JEDLIČKA” (ZFMK). ***C.suensoni***, **paratypes**: 1 male, “China, Hangchow 30°18'N, 120°07'E, 9.X.1921 Elgin Suenson leg.” (NHMW). 1 male, “China, Tien Mu Shan 30°23'N, 119°37'E, 19.VI.1937”, “paratypus”, “Callida (Callidiola) suensoni m. det. Kirschenhofer 83” (NHMW).

##### Notes on the type material.

***C.fukiensis***: This species was originally described based on a single specimen (holotype by monotypy) from “Kuatun, Fukien” (= Guadun, Fujian) deposited in ZFMK (Jedlička 1953). We examined this specimen through photograph (Fig. 1) thanks to the help of Dr Dirk Ahrens, the curator of ZFMK. We examined five further specimens in Jedlička’s collection (NMPC) subsequently identified by himself as *C.fukiensis*, not belonging to the type series. Three of them are corresponding to the holotype (see material examined below), while the other two actually belong to a different species, *C.klapperichi* Jedlička and *C.jelineki* Casale & Shi, respectively.

***C.suensoni***: This species was described based on the male holotype and eleven paratypes, with type locality: China, Tien-Mu-Shan (= Tianmushan, Zhejiang), 30°23'N, 119°37'E. We did not examine the holotype deposited in ZMUC, but the two examined paratypes (one of them from the type locality) plus one additional male specimen from the type locality, and the original description with male genitalia illustrations (Kirschenhofer, 1986) are enough to recognize this species as identical to *C.fukiensis*. It is noticeable that the original literature erroneously reported that this species has two apical setae on the abdominal sternite VII in males, and four in females. But actually it has four (two on each side) setae in males, and usually six (three on each side) in females.

##### Taxonomic remarks.

Jedlička (1953) described *Calleidafukiensis* as an “aberration” of *Calleidaonoha* Bates, 1873. Later Jedlička (1963) treated this taxon as a distinct species and provided a brief description. According to the International Code of Zoological Nomenclature, *C.fukiensis* Jedlička, 1953 is an unavailable infrasubspecific name, but *C.fukiensis* Jedlička, 1963 is available, whereas this name has been later ignored in recent catalogues (Lorenz 2005; Kabak 2017).

From the examination of abundant material for this species, including the type material cited above, we proved that the morphological features of these two taxa are coincident. So we treat *C.suensoni* Kirschenhofer, 1986 as a junior synonym of *Calleidafukiensis* Jedlička, 1963.

##### Non-type materials examined.

**Fujian**: 1 female, “Kuatun. Fukien. China. 27.6.46. (Tschung Sen.)” [type locality], “*fukiensis* Jedl. Det. Ing. Jedlička” (NMPC). 1 male, “Fujian, Chong’an, Xingcun, Sangang, 740 m”, “1960.V.17, leg. Pu Fuji” (IZAS). 1 male, “Fujian, Chong’an, Xingcun, Sangang, 740 m”, “1960.VII.18, leg. Pu Fuji” (IZAS). 1 female, “Fujian, Chong’an, Xingcun, Sangang, 900 m”, “1960.VI.26, leg. Zuo Yong” (IZAS). 1 male, “Fujian, Chong’an, Xingcun, Sangang, 740 m”, “1960.VI.24, leg. Zuo Yong” (IZAS). 1 female, “Fujian, Chong’an, Xingcun, Sangang, 740 m, light trap”, “1960.VI.30, leg. Zhang Yiran” (IZAS). 1 female, “Fujian, Chong’an, Xingcun, Sangang, 740 m”, “1960.VI.28, leg. Zhang Yiran” (IZAS). 1 female, “Fujian, Chong’an, Xingcun, Sangang, 720 m”, “1960.V.7, leg. Jiang Shengqiao” (IZAS). 1 female, “Fujian, Chong’an, Xingcun, Sangang, 720 m”, “1960.VI.24, leg. Jiang Shengqiao” (IZAS). 1 male, “Fujian, Chong’an, Xingcun, Tongmuguan, 740–850 m”, “1960.VII.24, leg. Zhang Yiran” (IZAS). 1 male, “Fujian, Jianyang, Huangkeng, Guilin, 290–310m”, “1960.VIII.4, leg. Pu Fuji” (IZAS). 1 female, “Fujian, Jianyang, Huangkeng, Dazhulan, 900–1170 m”, “1960.VII.5, leg. Pu Fuji” (IZAS). 1 female, “Fujian, Jianyang”, “1965.V.23, leg. Li Hongxing” (IZAS). 1 female, “Fujian, Jianyang, Guilin, 1979.VII.14, leg. Chen Chong” (IZAS). 1 female, “Wuyishan, Niyang, 570 m”, “1997.VIII.2, leg. Zhang Youwei” (IZAS). 1 female, “Fujian, Wuyishan, Pikeng, 520 m”, “1997.VII.14, leg. Wang Jiashe” (IZAS). 1 female, “Fujian, Wuyishan, Pikeng, 520 m”, “1997.VII.21, leg. Wu Yanyu” (IZAS). 1 female, “Wuyishan, Xianfengling, 1200 m”, “1997.VI.26, leg. Wu Yanyu” (IZAS). 1 female, “CHINA, Fujian Province, Wuyi Shan, ca860 m, 27°74'N, 117°66'E9.VI.2001, leg. J. Cooter” (CCA). 1 male, “Fukien. Shaowu. Chine. 1937. Klapperich”, “*fukiensis* n. sp. det. Ing. Jedlička” (NMPC). 1 female, “Fujian, Jiangle, 1985.X.9, leg Zeng Fanchun” (IZAS). 1 male, “Fujian, Jiangle, Longqishan”, “1991.V. 30, leg. Liu Hong” (IZAS). 1 female, “Fujian, Jiangle, Longqishan, Yujiaping, 800 m”, “1991.V. 28, leg. Li Hongxing” (IZAS). 1 female, “Fujian, Jiangle, Longqishan, 600m”, “1991.VI. 27, leg. Yang Longlong” (IZAS). 1 male, “Fujian, Jiangle, Longqishan”, “1991.V. 16, leg. Li Wenzhu” (IZAS). **Zhejiang**: 1 male, “Tiemushan, July, 19, 35” (IZAS). 1 female, “Tiemushan, August, 18, 35” (IZAS). 1 female, “T’ienmo Shan, 20.9.1953” (IZAS). 1 male “China, Tien Mu Shan 30°23'N, 119°37'E, 17.VI.1937, Elgin Suenson leg.” (CRS) (as *C.suensoni*). 1 male, “Shaohing, July, 12, 35” (IZAS). **Jiangxi**: 1 female, “Jiangxi, Ruijin, Baying”, “1980.VIII.16” (IZAS). 1 male, 2 females, “Jiangxi, Jiulianshan”, “1979.IX.30, leg. Yu Peiyu” (IZAS). **Henan**: 2 males, “ Henan, Luoshan, Dongzhai, 2005.VII.14–15, leg. Gao Chao, Wang Jiliang” (HBUM). **Hubei**: 1 female, „Hubei, Hefeng, 1989.VII.28, 870m, leg. Li Yongkun“ (IZAS). 1 female, “Hubei, Wudangshan, Laoying, 2004.IX.7, leg. Zhang Zhisheng, Chen Huiming” (HBUM). 1 female, “Hupeh Prov. China. River by city wall of Hwangmei. Hwang-mei Distr. 1933.VIII.3, leg. Y.W. Djou” (SYUM). **Hunan**: 1 female, “CHINA, Hunan Prov., Shimen Country, Hupingshan”, “2008.6.19–25 d, leg. Tang Guo” (IZAS). 1 male, “Hunan, Yongshun Coun., Shanmuhe forestry centre, 600m”, “1988.VIII.4, leg. Wang Shuyong” (IZAS). 1 female, “Hunan, Yongshun Coun., Shanmuhe forestry centre, 600m”, “1988.VIII.6, leg. Wang Shuyong” (IZAS). 1 male, “Hunan, Yongshun Coun., Shanmuhe forestry centre, 600–820 m”, “1988.VIII.7, leg. Wang Shuyong” (IZAS). 1 female, “Hunan, Yongshun County, Shanmuhe forestry centre, 600 m”, “1988.VIII.5, leg. Zhang Xiaochun” (IZAS). 2 males, 1 female, “Hunan, Yuanling, Jiemuxi, 2004.VIII.2–11, leg. Wang Jianfeng, Wang Jiliang” (HBUM). 1 female, “Hunan, Jianghua Coun., Sanjiangwei”, “1978.VI., leg. Peng Jianwen, Yin Shicai” (IZAS). **Guangdong**: 1 male, 1 female, “Guangdon, Nanling, leg. Wang L.” (SCAU). 1 female, “Guangdong, Huaiji” (SCAU). 1 female, “Guangdong, Lianxian”, “1965.VI.27, leg. ZhangYouwei” (IZAS). 1 female, “Guangdong, Shixing, Chebaling, Zhangdongshui; sweep net, 24.72320N, 114.25640E”, “354m, 2008.VII.27; day, leg. Tang Guo” (IZAS). **Guangxi**: 1 female, “Guangxi, Xing’an, Tongren”, “1985.VII.2, leg. Liao Subai” (IZAS). 1 female, “Guangxi, Xing’an, Jinshi, 2005.VII” (IZAS). 1 male, “Guangxi, Xing’an, Jinshi, 2007.VII” (IZAS). 1 female, “Guangxi, Xing’an, Mao’ershan” (IZAS). 1 male, 1 female, “Guangxi, Longsheng, Sanmen, 300 m”, 1963.VI.27, leg. Wang Chunguang” (IZAS). 1 female, “Guangxi, Longsheng, Sanmen, 300 m”, 1963.VI.28, leg. Shi Yongshan” (IZAS). 1 male, “Guangxi, Lingchuan, Lingtian, 225 m, 1984.VI.3” (IZAS). 1 female, “Guangxi, Lingchuan, Lingtian, 240, 1984.VI.6”, “8417” (IZAS). **Guizhou**: 2 males, 2 females, “Guizhou, Yanhe, Mayanghe, Daheba, 300m, 2007.IX.17–30, leg. Liu Ye” (IZAS). 2 males, “Guizhou, Yanhe, Daheba, 450–700m, 2007.VI.5–12, leg. Song Qiongzhang” (IZAS). 1 female, “Guizhou, Yanhe, Daheba, 450–700m, 2007.VI.5–12, leg. Zhang Pei” (IZAS). 1 male, “Guizhou, Jiangkou, Fanjingshan, 550m”, “1988.VII.13, light trap, leg. Yang Xingke” (IZAS). 2 females, “Guizhou, Daozhen, Yuheba, 2004.V.25, leg. Yu Yang” (HBUM). 2 males, 1 female, “Guizhou, Daozhen, Xiannüdong, 2004.VIII.24–26, leg. Yang Xiujuan, Hua Huiran” (HBUM). **Shaanxi**: 1 female, “China, Shaanxi, Foping, Changjiaoba, Shangshawo; 33.59716N, 108.01366E; 33.59212N, 108.02235E”, “1107–1215m, 2007.5.29, day, leg. Shi Hongliang” (IZAS). 1 female, “Shaanxi, Foping, 890 m, 1999.VI.26, leg. Zhang Youwei” (IZAS). **Shanxi**: 1 male, “Swan-ping. Mongolei.”, “ex Orig. Saml. J. Breit Wien”, “Museum J. Frey. Tutzing”, “*fukiensis* Jedl. Det. Ing. Jedlička” (NMPC).

##### Diagnosis.

With the character states of the *Calleidadiscoidalis* species group, but different from all other known species by the combination of the following features: (1) elytra metallic bluish green to bluish purple; (2) antennae and femora distinctly bicolor: basal three antennomeres reddish, the following darkened, femora with basal half reddish, apical blackish; (3) elytral basal border only extended from the shoulders to interval 4; (4) abdominal sternite VII with four setae in males (two on each side), six setae in females (three on each side, in some specimens with one additional seta on one side). This species is unique amongst all known Asiatic *Calleida* species by its incomplete elytral basal ridge. From all other species of the *C.discoidalis* group, and from all other *Calleida* species in China, *C.fukiensis* can be easily distinguished by its bluish elytra and bicolored femora.

##### Description.

*General features* as in Fig. 2. Small to medium- sized: L = 7.7–9.1 mm.

*Colour*: Head, pronotum and scutellum reddish orange to maroon; palpomeres and apex of mandible dark brown to blackish; apical palpomere light yellow at apex; the basal three antennomeres, and base of fourth yellow reddish, distal antennomeres dark brown, the second to fourth antennomeres a little darkened in some specimens; elytra bluish green to bluish purple, with marked metallic reflection and sutural area darker; epipleura metallic dark blue; ventral side orange yellow; legs yellowish to reddish brown, with apical third of femur and base of tibia blackish; tarsomeres dark brown on the dorsal side.

*Lustre and microsculpture*: Head and pronotum shiny, with highly effaced microsculpture; elytra shiny, with fine but distinct isodiametric reticulate microsculpture and marked metallic lustre.

*Head*: Smooth or very finely and sparsely punctate; frons with oblique, faint wrinkles at sides; supraorbital furrows deep, interrupted before the level of hind edge of eyes; genae longer than the half length of eyes; temporae swollen, gradually narrowed towards the neck constriction; apical labial palpomere securiform, truncate at apex in males, less tumid and not truncate in females; mentum with lateral lobes triangular, the inner margins oblique; median tooth obtuse, with two short setae, inserted in the middle part of the tooth.

*Pronotum*: Transverse-cordiform (ratio PW/PL = 1.13–1.18), greatest width at approximate anterior third; lateral margins arcuate near the middle, slightly sinuate anteriorly to the posterior angles; posterior angles almost rectangular or slightly acute in some specimens; lateral expansions narrow; disc slightly convex, with sporadic transverse wrinkles and fine punctures.

*Elytra*: Elongate (ratio EL/EW = 1.64–1.68), with basal ridge only extended from the shoulders to the fourth interval; striae moderately deep, finely punctate, punctures gradually weakened in the apical part of elytra; intervals flat, finely and sparsely punctate; the eighth interval slightly tumid near apex; umbilicate series of composed of 15 pores along stria 8; apical truncation straight or slightly concave; outer apical angle thickened but not angulate.

*Ventral side*: Prosternum, lateral area of metasternum, and metepisterna finely pubescent; abdominal sternites with sparse and short accessory setae; sternite VII with four setae in males (two on each side), six setae in females (seldom with one additional seta on one side) (Casale and Shi 2018: figs 2, 5); apical margin of abdominal sternite VII distinctly notched in males, straight in females.

*Male genitalia* (Figs 6–8): Median lobe of aedeagus slightly bent, its middle part strongly expanded in dorsal view; dorsal and ventral margins slightly curved in lateral view; apical orifice pleuropic left; apical lamina flat and rounded, apex expanded and thickened; endophallus with two chitinized copulatory pieces, located in the middle area near the left lateral margin, long and narrow, close to each other at base, V-shaped; left paramere depressed on the dorsal side; right paramere not emarginate at apex.

*Female genitalia* (reproductive tract Fig. 3 and gonocoxa Figs 4, 5): Spermatheca digitiform, as long as the pedicel, with long basal projection; surface faintly and finely whorled; spermathecal pedicel with basal half expanded, apical half slender, with an indistinct apical protuberance; spermathecal gland duct laterally inserted at base of the projection, long and slender, about twice length as spermatheca; glandular area slightly incrassate, about two thirds as long as gland duct, base with very weakly defined atrium. Gonocoxite II sub-rectangular, as long as three times the basal width; base slightly wider than apex; inner margin with several setae, extending from the basal third to apex; outer margin distinctly curved at middle, only slightly setose in the apical third; apex obliquely truncate, with irregular membranous extension on the outer part.

##### Geographical distribution and habitat.

**Map 1.**
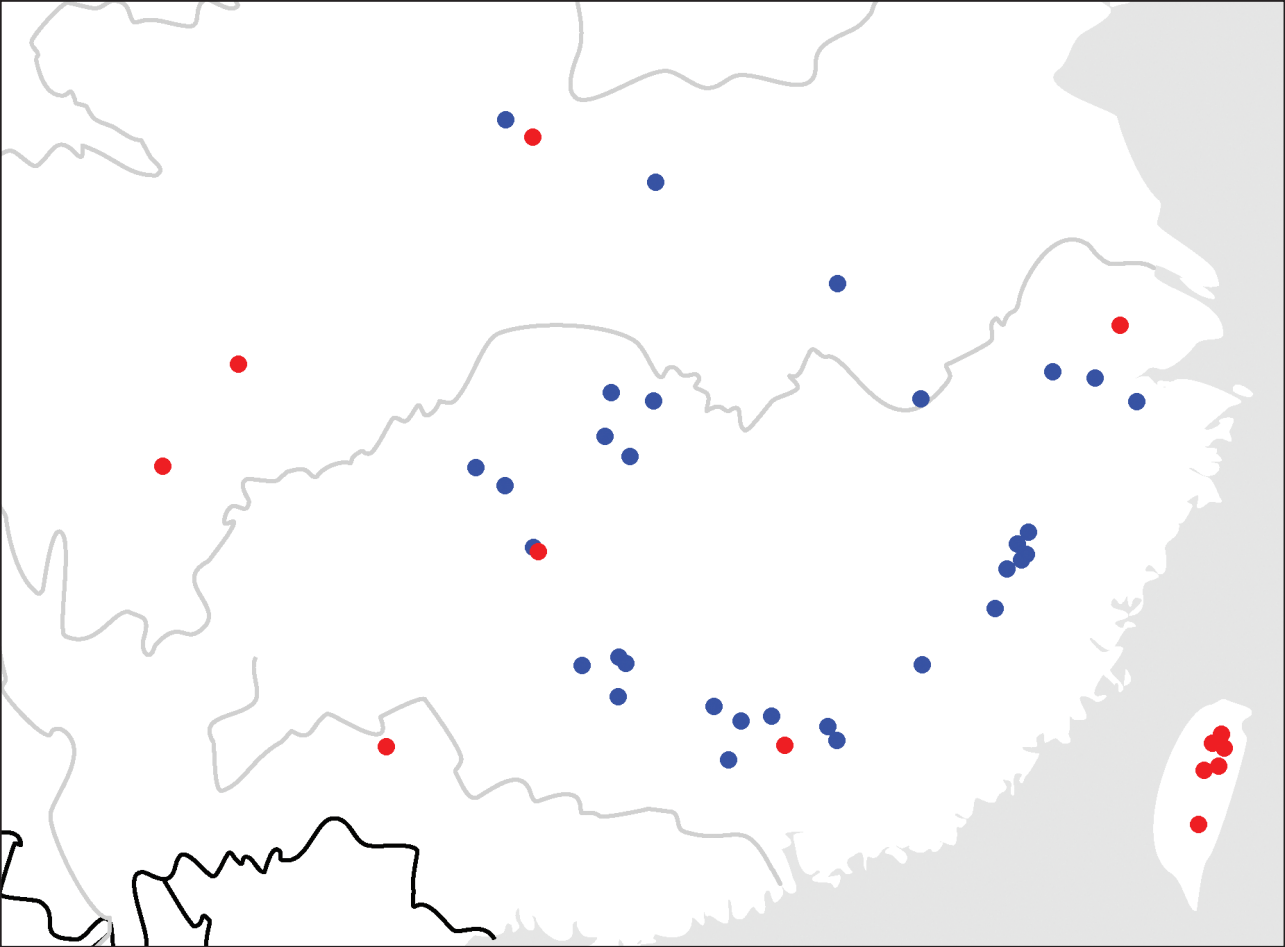
Distributions for species in the *C.discoidalis* group: *C.fukiensis* Jedlička (blue); *C.piligera* sp. n. (red).

Endemic to China, but widespread and known from several provinces of South China: Zhejiang, Fujian, Jiangxi, Henan, Hubei, Hunan, Guangdong, Guangxi, Guizhou, Shaanxi. *C.fukiensis* is one of the most widely spread *Calleida* species in China (Map 1).

We examined one male in NMPC labeled as “Swan-ping. Mongolei”, referring to Shuo-ping Fu, an old name for the region around Youyu county (39.98N, 112.47E, 1300 m) in northernmost Shanxi. This locality is far from all other confirmed localities of this species. We consider it to be a dubious record and was not included in the distribution map.

This species was mainly found in evergreen broad-leaf forest of southern China. Some specimens were collected on vegetation or by light trap.

#### [2] Calleida piligera

Taxon classificationAnimaliaColeopteraCarabidae

Shi & Casale sp. n.

http://zoobank.org/DB98BF02-9165-4B11-A8B9-CCE0E9420930

Figs 9–18 , Map 1

##### Type locality.

Taiwan, Taoyuan county, Siling (24.65N, 121.42E, 1100 m).

##### Type materials.

**Figures 9–18.**
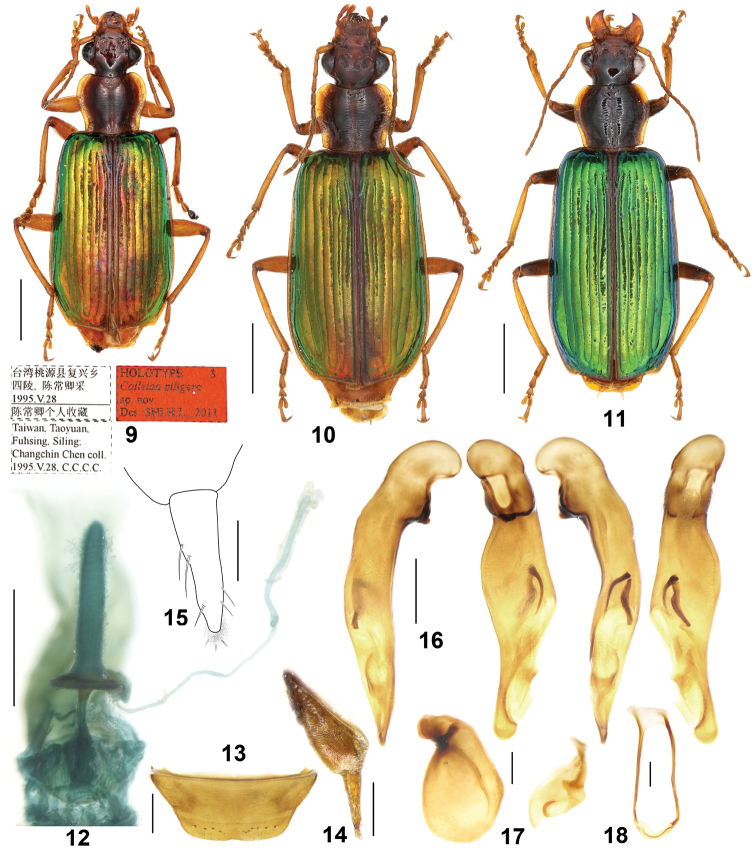
*Calleidapiligera* sp. n. **9** Habitus and labels of holotype (male, Taiwan, NMNST) **10** Habitus (female paratype, Taiwan, IZAS) **11** Habitus (female paratype, Taiwan, IZAS) **12** Female reproductive tract (paratype, Taiwan, IZAS) **13** Female sternite VII (paratype, Taiwan, IZAS) **14** Gonocoxa (paratype, Taiwan, IZAS) **15** Gonocoxite II (paratype, Taiwan, IZAS) **16** Median lobe of aedeagus, right-lateral, ventral, left-lateral, and dorsal views (holotype) **17** Left and right parameres of aedeagus (holotype) **18** Male sternite IX (holotype). Scale bars: 2.0 mm (**9–11**), 0.5 mm (**12, 13, 16, 18**), 0.2 mm (**14, 17**), 0.1 mm (**15**).

**Holotype**, male, “Taiwan, Taoyuan, Fuhsing, Siling; leg. Changchin Chen, 1995.V.28, C.C.C.C.” (NMNST, Fig. 9). **Paratypes** (a total of 23 specimens): **Taiwan**: 2 males, 1 female, same data as holotype (CCA, CCCC). 1 male, 1 female, “Taiwan, Nantou, Ren’ai, Sungkang, 2000 m, leg. Chinchi Lo, 1995.VI.23” (CCCC). 1 female, “Taiwan, Nantou, Ren’ai, Sungkang; 2000 m; leg. Chinchi Lo, 1995.VI.2” (CCCC). 1 female, “Taiwan, Nantou, Ren’ai, Sungkang, leg. Changchin Chen, 1994.VIII.16”(CCCC). 1 male, “Taiwan, Taitung, Hsiang Yang; leg. Wensin Lin; 2008.IV.26N” (CCCC). 2 females, “Taiwan, Ilan, Tatung, Siyuan, leg. Changchin Chen, 1998.V.30”(CCCC). 1 female, “Taiwan, Hsinchu, Jianshi, leg. Changchin Chen, 1994.VI.11” (CCCC). 3 females, “Taiwan, Hualien, Sioulin, Bilyu Scared Tree, leg. Changchin Chen, 1995.V.2” (CCA, CCCC). **Shaanxi**: 1 male, “China, Shaanxi, Ningshan, Huoditang; 33.43368N, 108.44747E”, “1538m, 2007.VI.2, beating, leg. Shi Hongliang” (IZAS). **Shanghai**: 1 female “Shanghai”, “Coll. Armitage”, “Museum Paris Coll. R. Oberthür, 1952” (MNHN). **Guangdong**: 1 male, “Guangdong, Shimentai N.R. leg. Tian M.Y” (SCAU). 1 female “Guangdong” (CCA). **Guangxi**: 1 male, “Guangxi, Tianlin, Cenwanglaoshan; 1200–1300 m; 2002.V.28, leg. Yang Xiujuan” (HBUM). **Guizhou**: 1 female, “Guizhou, Fanjingshan, Heiwanhe; 1200 m; 2002.VI.4; leg. Song Qiongzhang” (IZAS). 1 male, “Guizhou, Fanjingshan, N27°89.962’, E108°70.826’; 1500–2000 m;”, “2008.VII.3, light trap; leg. Li Yu, B08L9135” (CCCC). **Sichuan**: 1 male “China, Sichuan, Qingchenghou Shan 70 km NW Chengdu 1500 m 6.–13.VIII.2010 S. Murzin” (ZSM). 1 female, “West Sichuan Gongga Shan (Moxi env.) 3000 m 22.V–10.VI.1993 leg. V. Beneš” (CPB).

##### Specific epithet.

In Latin, *piliger* means setose. The specific name is referred to the remarkable high number of setae on the abdominal sternite VII in both sexes.

##### Diagnosis.

With the character states of the *Calleidadiscoidalis* species group, but differing from all other known species by the combination of: (1) elytra uniformly metallic green or cupreous green, without discal patch; (2) head and pronotum brownish, without metallic lustre; (3) abdominal sternite VII with eight or more setae in both males and females; (4) pronotum only with a few fine punctures along the median furrow.

Compared with *Calleidafukiensis*, the new species differs by the complete elytral basal ridge that reaches the parascutellar stria, the much higher number of setae on abdominal sternite VII, and the different body colour.

In this species group, the new species is somewhat similar to *C.yunnanensis* sp. n., but differs from that species by the elytra lacking any trace of discal reddish patch, and males with more than eight setae on the terminal ventrite. Amongst the other *Calleida* species of China, the new species is similar to *C.klapperichi* Jedlička, 1963 in general appearance, but can be readily distinguished by the number of setae on abdominal sternite VII.

##### Description.

*General features* as in Figs 10, 11. Medium-sized: L = 9.5–11.0 mm.

*Colour*: Head dark brown, mouth part and antennae yellowish brown; pronotum dark brown, with lateral expansions yellowish; scutellum reddish brown; elytra uniformly metallic green, usually with cupreous reflection, without reddish discal patch; elytral suture and lateral margins yellowish brown; epipleura yellowish brown; ventral side and legs yellowish brown, femora darkened in some specimens.

*Lustre and microsculpture*: Head without microsculpture; pronotum mostly without distinct microsculpture, except some fine transverse meshes near the discal transverse wrinkles, and faint isodiametric meshes on the middle part of the basal area; elytra with distinct microsculpture in an isodiametric mesh.

*Head*: Moderately convex; frons with a few very fine punctures and oblique wrinkles laterally, distinct or very faint; supraorbital furrows deep, extended to the level of posterior edge of eyes; temporae not swollen, gradually narrowed towards the neck; genae shorter than the half length of eyes; antennae reaching the basal fifth of elytra; terminal labial palpomere strongly securiform with truncate apex in males, only slightly expanded in female; mentum lateral lobes with outer margins slightly arcuate, inner margins oblique, mentum tooth near triangular, rounded at apex, with two short setae inserted in middle part of the tooth.

*Pronotum*: Transverse (ratio PW/PL = 1.19–1.28), with its maximum width at about the anterior third; lateral margins gradually arcuate near the middle, straight or slightly sinuate before the posterior angles which are obtusely rounded; lateral expansions widened, explanate in front; disc slightly convex, with distinct transverse wrinkles and with a few fine punctures along only the median furrow; median furrow distinct, but interrupted before both the anterior and posterior margins.

*Elytra*: Elongate (ratio EL/EW = 1.65–1.75), with basal border complete, reaching the parascutellar stria; striae distinct, finely punctate, the punctures gradually weakened in the apical part; intervals slightly convex, finely and sparsely punctate; intervals without additional setigerous pores; third to fifth intervals slightly depressed at the basal fourth; eighth interval slightly tumid at apex; umbilicate series of 15–16 pores along eighth stria; apical truncation slightly concave; lateral margins distinctly thickened at the outer apical angles, which are obtusely rounded.

*Ventral side*: Prosternum, lateral area of metasternum, and metepisterna with fine pubescence; abdominal sternites with dense and long accessory setae; abdominal sternite VII with 8–12 setae in both male and female (four to six on each side) (Fig. 13), usually not regularly arranged in a row; apical margin of abdominal sternite VII distinctly notched in male, straight or slightly emarginate in female.

*Male genitalia* (Figs 16–18): Median lobe bent, pleuropic left; in ventral aspect middle portion strongly widened, right lateral margin strongly sinuate; apical lamella flat, a little shorter than basal width, fully rounded at apex. Endophallus with two chitinized copulatory pieces close to left lateral margin of the median lobe, their apex adjacent to the base of the apical orifice, close to each other at base, V-shaped, the ventral one sinuate and acerate, the dorsal one a little wider. Left paramere dorsally depressed, about 1.5 times as long as wide; right paramere not emarginate.

*Female genitalia* (reproductive tract Fig. 12 and gonocoxa Figs 14, 15): Spermatheca digitiform, twice longer than the pedicel, surface not whorled, base with an evident basal sclerotized plate (annulus receptaculi); spermathecal pedicel with basal part a little expanded, apical part slender; spermathecal gland duct laterally inserted at the basal surface of the plate, very long and slender, a little longer than spermatheca; glandular area slightly incrassate, a little shorter than gland duct, base with very small atrium. Gonocoxite I moderately wide, with microsculpture; gonocoxite II subulate, apex sharp, length about three times as basal width; inner margin with several setae, extending from the basal third to apex, outer margin only slightly setose in the apical third; both outer and inner margins straight; apical margin with membranous extension and short setae.

##### Geographical distribution and habitat.

Widely distributed in several provinces of south China: Taiwan, Shaanxi, Shanghai, Guangdong, Guangxi, Guizhou and Sichuan (Map 1). Common at mid-high elevations (ca 2000m) in Taiwan, but apparently rare in Chinese continental provinces. This species was mainly found in evergreen broad-leaf forest. Some specimens were collected by beating from vegetation or in light trap.

##### Remarks.

From many important morphological aspects, *C.piligera* sp. n. is very peculiar in the *C.discoidalis* species group: (1) the base of spermatheca has an evident annulus receptaculi, surface not whorled; in contrast spermatheca is without such structure and surface more or less whorled in all other known species; (2) terminal ventrite with four or more setae on each side in males; in contrast usually with two (exceptionally three) setae on each side in males for all other known species; and (3) gonocoxite II subulate, apex narrowed and sharp in contrast to gonocoxite II with apex more or less oblique truncated in all other known species. The similarities of spermatheca lead us to hypothesize a relationship of the new species to the *C.terminata* species group, in which all known species have spermatheca with annulus receptaculi and without whorled surface (Casale and Shi 2018: figs 55, 60). But, in several other aspects, such as shape of the male genitalia, setation on the terminal ventrite, and shape of elytral apical outer angles, the new species does not accord with the *C.terminata* group at all. As discussed above, the *C.discoidalis* group is probably not monophyletic. As we just erected it as a group of convenience to accommodate species with similar multiple setation of the abdominal sternite VII and male genitalia character, the question of species relationship must await a future answer.

In most specimens, the elytra are metallic green, with distinct cupreous reflection (Figs 9, 10), but in two females from Songgang (Taiwan: Nantou), the elytra are vivid green, without cupreous reflection (Fig. 11).

#### [3] Calleida cochinchinae

Taxon classificationAnimaliaColeopteraCarabidae

Casale & Shi sp. n.

http://zoobank.org/D4CD66E9-39FF-4DE6-BFE6-CA1C3448DF5B

Figs 19–21 , Map 2

##### Type locality.

S. Vietnam: “Cochinchina”.

##### Type material.

**Figures 19–21.**
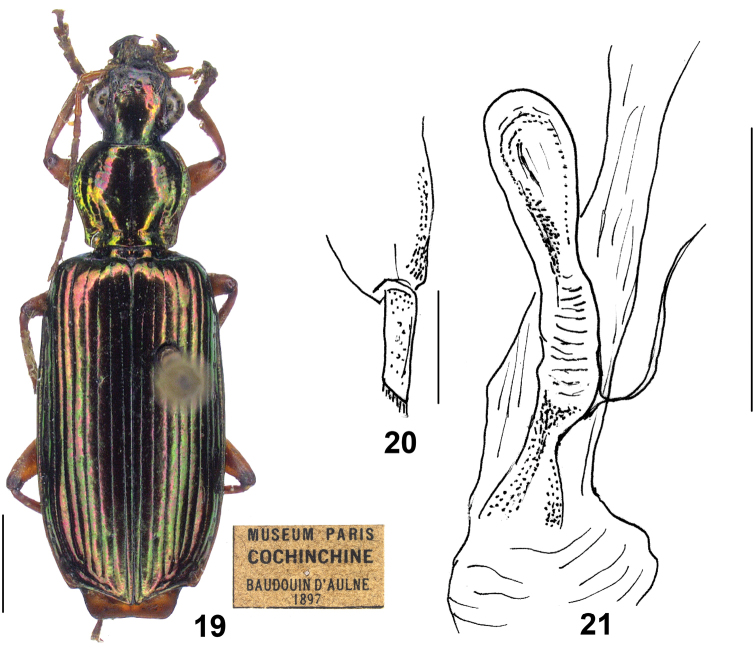
*Calleidacochinchinae* sp. n. **19** Habitus and labels of holotype (female, Cochinchine, MNHN) **20** Gonocoxite II (holotype) **21** Female reproductive tract (holotype). Scale bars: 2.0 mm (**19**), 0.2 mm (**20**), 0.5 mm (**21**).

**Holotype**, female, “MUSEUM PARIS Cochinchine, Baudouin d’Aulne 1897”, “Calleida cochinchine” (MNHN, Fig. 19).

##### Specific epithet.

The name is derived from the former name of the southern province of Vietnam of the former French empire (1862–1954), as indicated in the original label of the holotype.

##### Diagnosis.

This brilliant new species is distinct amongst all Asiatic *Calleida* species for: (1) abdominal sternite VII with five setae on each side in females (males unknown); (2) head, pronotum and ventral side uniformly metallic green, with marked cupreous reflection; (3) elytra rather elongate; (4) elytral apical margin strongly concave, with margins thickened at the outer apical angles but not angulate. *C.cochinchinae* can be easily distinguished from all other species in the *C.discoidalis* species group by its special coloration, but might be confused with *C.viet* Casale & Shi, also from South Vietnam, which has similar completely metallic greenish dorsal surface. Different from that species, the new species has a more elongate shape (EL/EW = 1.84, contrasting to 1.68 in *C.viet*), less prominent elytral outer apical angles, and multisetose abdominal sternite VII (males unknown, but supposed multisetose also).

##### Description.

*General features* as in Fig. 19. Medium to large- sized: L = 12.0 mm (female holotype). Body elongate and slender, dorsal and ventral side completely metallic.

*Colour*: Head, pronotum, elytra (epipleura included), and ventral side uniformly metallic green with marked cupreous reflection, more evident at the elytral base and apex; clypeus and labrum blackish, palpomeres dark brownish with yellowish apex for the terminal segments; antennae and legs reddish yellow; legs with faint metallic green reflection; apex of femora, and all tarsomeres dark brownish.

*Lustre and microsculpture*: Dorsal surface rather shiny and polished; head and pronotum with vanished microsculpture; elytra with faint but distinct microsculpture in isodiametric meshes.

*Head*: Slightly convex, almost impunctate; frons with very faint transverse wrinkles at sides; supraorbital furrows moderately deep, vanished anteriorly to the half of the inner edge of eyes; temporae barely swollen, narrowed towards the neck; genae short, as long as the half length of eyes. Antennae short, only slightly exceeding the humeral angles of elytra. Terminal labial palpomere markedly dilated in females (probably securiform in males); mentum lateral lobes with outer margins straight, the inner margins oblique; mentum tooth obtusely truncate at apex, with two short setae inserted at the middle part of tooth.

*Pronotum*: Roundish-cordate (ratio PW/PL = 1.14), with its maximum width at about the anterior third; lateral expansions moderately wide; lateral margins reflexed and arcuate at the middle, distinctly sinuate before the posterior angles which are obtuse, not pointed at apex. Disc slightly convex, with deep transverse wrinkles and with a few punctures along the median furrow; median furrow deep, widened at base and reaching the posterior margin.

*Elytra*: Elongate (ratio EL/EW = 1.84), with basal ridge complete, extended to the parascutellar stria; striae deep, finely punctate; intervals flat, very finely and sparsely punctate; intervals 7–8 moderately tumid at apex; umbilicate series of 13 (right elytron) to 15 (left elytron) pores along the eighth stria; apical truncation oblique, markedly concave, margins thickened at outer apical angels, which are obtusely prominent, not angulate.

*Ventral side*: Glabrous; in the female holotype, abdominal sternite VII with five setae on each side, apical margin almost straight, slightly excised at middle. Male sternum unknown.

*Male genitalia*: Unknown.

*Female genitalia* (reproductive tract Fig. 21 and gonocoxa Fig. 20): Spermatheca slightly dilated to apex, a little longer than the pedicel, base without projection; surface distinctly whorled; spermathecal pedicel markedly dilated at the basal two thirds, slender in the apical third; spermathecal gland duct laterally inserted, rest portion not examined (damaged in the only available individual). Gonocoxite I wide; gonocoxite II subulate, elongate, a little narrowed to apex, about four times as long as wide at base; inner margin setose in the apical fourth; both outer and inner margins straight; apex obliquely truncate, with membranous setose extension.

##### Geographical distribution and habitat.

**Map 2.**
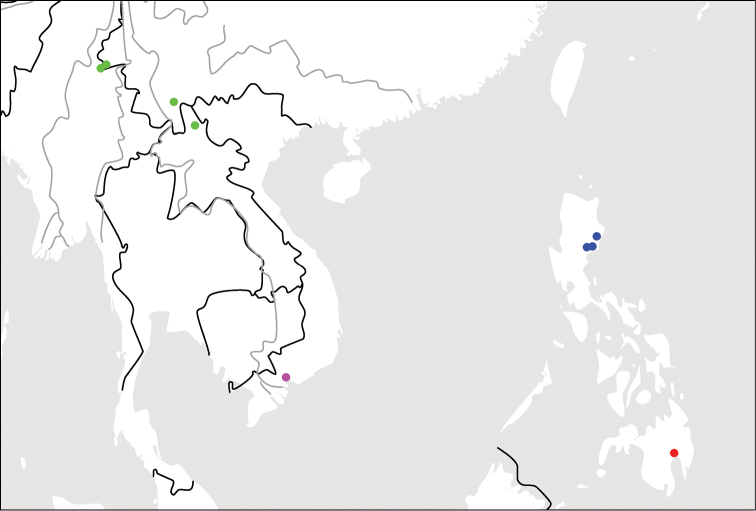
Distributions for species in the *C.discoidalis* group: *C.cochinchinae* sp. n. (magenta); *C.yunnanensis* sp. n. (green); *C.discoidalis* Heller (blue); *C.luzonensis* sp. n. (red).

Only known from the single holotype female from Vietnam: “Cochinchina”, without further information on the type locality in the label (Fig. 19 and Map 2). No data are available on the habitat, which should be located in tropical forests of the region.

##### Remarks.

From the rather elongate habitus and peculiar female genital characters, *C.cochinchinae* sp. n. is different from all other known species in the *C.discoidalis* species group, but surprisingly accords with the *C.lativittis* species group. Amongst all examined species of Asiatic *Calleida*, only *C.cochinchinae* and species in the *C.lativittis* group (two species with female genitalia examined of three species of the group; Casale and Shi 2018) have the following character combinations of the female reproductive tract: spermatheca without basal projection or plate, and the spermathecal gland duct inserted on the lateral side of spermatheca. Thus, we inferred a relationship of the new species with the *C.lativittis* species group rather than to other species of the *C.discoidalis* group, which was erected as a “group of convenience” to accommodate species with similar multisetose abdominal sternite VII. This relationship was also supported by our phylogenetic analysis (Fig. 43).

#### [4] Calleida yunnanensis

Taxon classificationAnimaliaColeopteraCarabidae

Shi & Casale sp. n.

http://zoobank.org/A9F123C3-A55D-44D3-AE29-6564D5D82A58

Figs 22–31 , Map 2

##### Type locality.

Yunnan, Pu’er city, Caiyanghe (22.60N, 101.12E, 1700 m).

##### Type materials.

**Figures 22–31.**
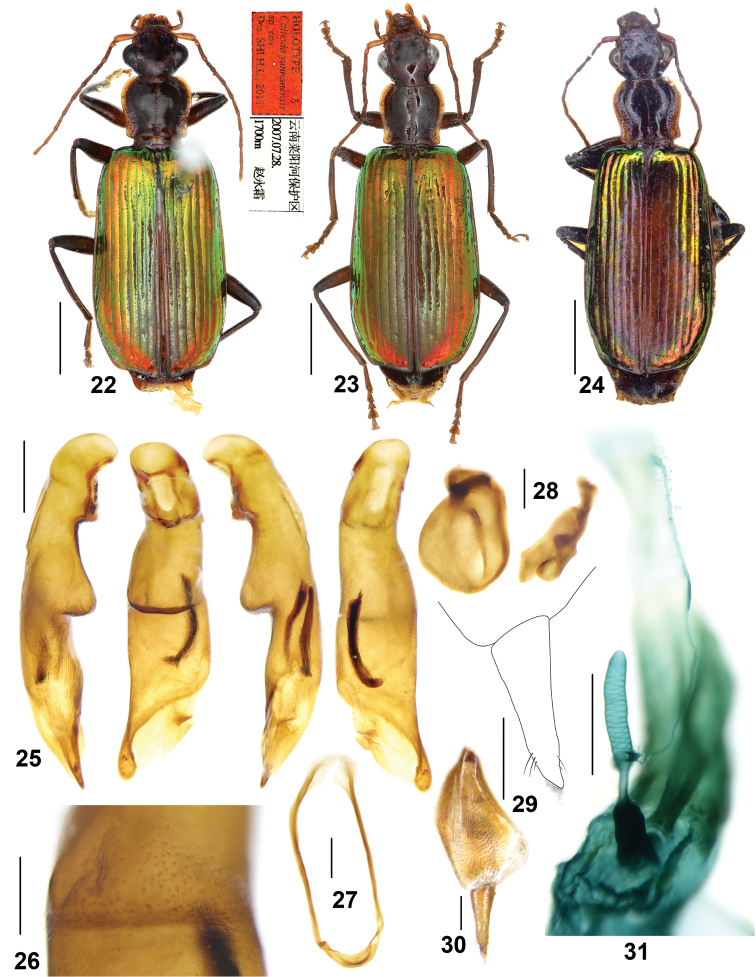
*Calleidayunnanensis* sp. n. **22** Habitus and labels of holotype (male, Yunnan, IZAS) **23** Habitus (female paratype, Yunnan, IZAS) **24** Habitus (female paratype, Laos, NHML) **25** Median lobe of aedeagus, right-lateral, ventral, left-lateral, and dorsal views (holotype) **26** Cuneiform projection on median lobe, ventral view (holotype) **27** Male sternite IX (holotype) **28** Left and right parameres of aedeagus (holotype) **29** Gonocoxite II (paratype, Yunnan, IZAS) **30** gonocoxa (paratype, Yunnan, IZAS) **31** Female reproductive tract (paratype, Yunnan, IZAS). Scale bars: 2.0 mm (**22–24**), 0.5 mm (**25, 27, 31**), 0.2 mm (**26, 28, 30**), 0.1 mm (**29**).

**Holotype**: Male, “Yunnan, Simao, Caiyanghe national reserve, 1700 m, 2007.VII.28, leg. Zhao Yongshuang” (IZAS, Fig. 22). **Paratypes** (a total of 8 specimens): **Yunnan**: 2 females, same data as holotype (IZAS and CCA). 1 female, “Yunnan, Simao, Caiyanghe national reserve, Liechang, 2009.III.7, leg. Zhu Xiaoyu” (CCCC). 1 male, “Yunnan, Ruili, Mengxiu, 2150 m, 2005.VIII.3, leg. Mao Benyong” (HBUM). 1 female, “Yunnan, Ruili, Nongdao, Dengga, 1000 m, 2006.VIII.1, leg. Liu Biao” (IZAS). 1 female, “Yunnan, Ruili, Nongdao, Dengga to Mafengshan, 23.95285N, 97.59808E – 23.94485N, 97.55647E”, “927–1207 m, 2009.VIII.10, leg. Shi Hongliang, beating” (IZAS). 1 female, “Yunnan, Ruili, Nongdao, Wudianshan, 920m, 2015-IX-11, beating on vegetation, Yang Xiaodong leg.” (CCCC). **Laos**: 1 female, “Haut Mekong. Pou Lan. 13.V.1918. R.V. de Salvaza”, “1961”, “Brit. Mus. 1921-89” (BMNH).

##### Specific epithet.

The name of the new species refers to its type locality: Yunnan, China.

##### Diagnosis.

With the character states of the *C.discoidalis* species group, but different from all other known species by the combination of: (1) elytra metallic green, with evident lustre of cupreous red or purple; (2) elytral discal reddish patch not well defined; (3) head, disc of pronotum and legs brownish, markedly darkened; (4) elytra with several very small additional setae on odd intervals; (5) abdominal sternite VII with two (seldom three) setae on each side in males, four or more in females; (6) median lobe of aedeagus with a cuneiform projection on the ventral side.

In this species group, *C.yunnanensis* sp. n. is somewhat similar to the two species (*C.discoidalis* and *C.luzonensis*) from the Philippines based on the presence of a reddish patch on elytral disc. But from the other two, *C.yunnanensis* can be distinguished by: (1) the darker color on head, pronotum, legs and ventral side; (2) the presence of cupreous reddish hue on elytra; (3) narrower pronotum and elytra; and (4) the characters of aedeagus which has a peculiar projection on the median lobe ventral surface.

In southern Yunnan, *C.yunnanensis* sp. n. is sympatric with *C.quadricollis* Straneo and one undescribed species belonging to the *C.splendidula* species group. These three species are similar in general features and coloration, but *C.yunnanensis* sp. n. can be readily distinguished by its higher number of setae on the terminal ventrite in both sexes.

##### Description.

*General features* as in Figs 22–24. Medium-sized: L = 9.4–9.8 mm.

*Colour*: Head dark reddish brown, vertex distinctly darker, approximate piceous; mouth parts and antennae yellowish brown, terminal palpomere with light yellow apex. Pronotum dark brown, with lateral expansions yellowish brown and disc slightly lighter along the median furrow; scutellum reddish brown. Elytra metallic green with marked cupreous reddish or purplish reflection, more evident in the apical area; metallic reflection gradually fainter toward the suture, forming a vague discal reddish brown patch almost without metallic lustre on the apical half or two thirds, not reaching elytral base, widest near elytral apical third or fourth, occupying the inner three or four intervals, sometimes the fifth interval also partly brownish; lateral margins, elytral suture, and epipleura brownish with faint metallic reflection. Ventral side reddish brown to dark brown, with metacoxae and adjacent metasternal area generally lighter; legs brown, femora gradually darker to apex, tibiae infuscate at base, tarsomeres yellowish brown.

*Lustre and microsculpture*: Dorsal surface moderately shiny and polished; head and pronotum with vanished microsculpture; elytra with faint microsculpture in isodiametric meshes, more evident in females.

*Head*: Slightly convex, almost impunctate; frons with oblique wrinkles aside, weakly defined or very faint; supraorbital furrows moderately deep, vanished at half of the inner edge of eyes; temporae moderately swollen, gradually narrowed towards the neck; genae longer than the half length of eyes; antennae reaching the basal fifth of elytra; terminal labial palpomere strongly securiform with truncate apex in males, also dilated but less so in females; mentum lateral lobes with outer margins straight, inner margins oblique; mentum tooth obtuse with apex truncate or slightly rounded, with two short setae inserted at the base of tooth.

*Pronotum*: Sub-quadrate (ratio PW/PL = 1.07–1.13), with its maximum width near anterior third; lateral expansions moderately wide; lateral margins arcuate at the middle, more or less sinuate before the posterior angles; posterior angles rectangular or obtuse, sometimes slightly pointed at apex; disc weakly convex, sometimes with very faint transverse wrinkles and a few punctures along the median furrow; median furrow distinct, but not reaching anterior nor posterior margins.

*Elytra*: Elongate (ratio EL/EW = 1.72–1.75), with basal border complete, extended to the parascutellar stria; striae distinct, finely punctate, with punctures gradually weakened in the apical part; intervals flat, finely and sparsely punctate; the third, fifth and seventh intervals each with more than 10 very small additional pores, slightly larger than interval punctures, each bearing one short seta, setae a little longer in the basal area of intervals; the eighth interval distinctly tumid near apex; umbilicate series of 15–16 pores along the eighth stria; apical truncation straight or very weakly concave; lateral margins distinctly thickened at the outer apical angles, which are obtusely rounded.

*Ventral side*: Mostly glabrous, lateral areas of prosternum at apex and metasternum with a few short setae; abdominal sternites sparsely pubescent. Abdominal sternite VII with two (the holotype bearing three setae on the right side) setae on each side in males, the inner seta placed a little forward; with four to six setae on each side in females, with the outer second seta placed a little forward; apical margin of abdominal sternite VII distinctly notched in males, straight in females.

*Male genitalia* (Figs 25–28): Median lobe of aedeagus pleuropic left, peculiarly shaped, with a conspicuous cuneiform projection near the middle of the ventral side (Fig. 25); apical surface of the projection bearing numerous fine setae (Fig. 26), almost perpendicular to the surface of the ventral margin; right lateral surface finely and longitudinally wrinkled before the apical orifice; apical lamina flat, a little longer than the basal width, rounded at apex. Endophallus with two chitinized copulatory pieces, close to the left lateral side of the median lobe, adjacent to each other at base, nearly V-shaped. Left paramere rounded, longitudinally depressed on the dorsal side, raised at base, about one and a half times longer than wide; right paramere slightly emarginate.

*Female genitalia* (reproductive tract Fig. 31 and gonocoxa Figs 29, 30): Spermatheca digitiform, not so straight as in the species treated above, nearly as long as the pedicel, with a stout triangular basal projection (much shorter than in *C.fukiensis*); surface distinctly whorled; spermathecal pedicel markedly dilated at the basal two thirds, apical third slender; spermathecal gland duct laterally inserted at base of the projection, very long and slender, about 1.5 times as long as spermatheca; glandular area barely incrassate, about same length as gland duct, base with atrium not protuberant. Gonocoxite I wide, with microsculpture; gonocoxite II narrow, approximate subulate, three times as long as wide at base; inner margin setose in the apical fourth; both outer and inner margins straight; apex obliquely truncate with membranous setose extension.

##### Geographical distribution and habitat.

Known from two localities of southern and southwest Yunnan, and northern Laos (Luang Namtha) (Map 2). In southern Yunnan, this species was found in a mountain rainforest and an evergreen broad-leaf forest at middle elevation (ca 1000 m). One specimen was collected by beating shrubs along a dirt path.

##### Remarks.

We examined one female from northern Laos (Fig. 24), labeled as “Haut Mekong. Pou Lan.” (R.V. de Salvaza) (BMNH) probably referring to Ban Phou Lan (20.555N, 101.001E, 890 m) in Luang Namtha Province. Different from other specimens in Yunnan, this specimen from Laos has the elytra almost completely greenish, only with very faint reddish hue, 12 setae in female abdominal sternite VII, and a little wider gonocoxite II. Besides the above minor differences, all characters including the female reproductive tract have no significant difference from the typical *C.yunnanensis* from Yunnan, China. So at moment we attribute this specimen to *C.yunnanensis*, although its distribution is a little distant.

#### [5] Calleida discoidalis

Taxon classificationAnimaliaColeopteraCarabidae

Heller, 1921

Figs 32–35 , Map 2

Callida discoidalis Heller, 1921: 529 (type locality: Philippines: Mindanao, Davao; holotype deposited in SMTD); Jedlička 1963: 436.

##### Type material examined.

**Figures 32–35.**
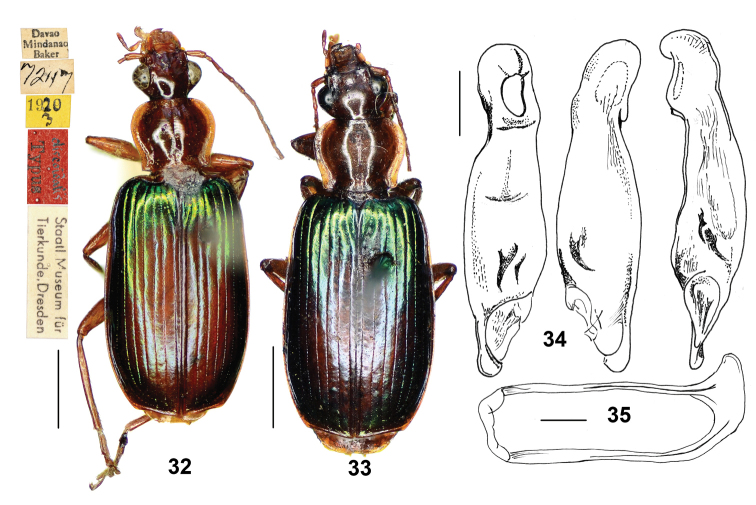
*Calleidadiscoidalis* Heller. **32** Habitus and labels of holotype (male, Mindanao, SMTD) **33** Habitus (female, Mindanao, NHML) **34** median lobe of aedeagus, ventral, dorsal, and left-lateral dorsal views (holotype) **35** Male sternite IX (holotype). Scale bars: 2.0 mm (**32, 33**), 0.5 mm (**34, 35**).

**Holotype**, male, “Davao Mindanao Baker”, “7247”, “1920/3”, “discoidalis. Typus”, “Staatl. Museum für Tierkunde. Dresden” (SMTD, Fig. 32).

##### Non-type materials examined.

1 female, “Davao Mindanao Baker”, “Ex Mus. Coll. Agric. Phil. Is.”, “Callidadiscoidalis Heller compared with type H.E.A.”, “H.E. Andrewes Coll. B. M. 1945-97.” (BMNH). 1 female, “Davao Mindanao Baker” (CCA, ex Straneo collection).

##### Diagnosis.

*C.discoidalis* is distinct within the *Calleidadiscoidalis* group for its evident reddish spot on the inner five or six intervals of the elytra, markedly contrasting with the lateral metallic bluish green intervals, and for having the head, antennae, pronotum, legs and ventral side completely reddish yellow. *C.discoidalis* is similar to *C.luzonensis* n. sp., from which is distinct by several characters stressed in the key to species (and see diagnosis of *C.luzonensis* below).

*C.discoidalis* could be confused with *C.splendidula*, sympatric in the Philippines, for their similar habitus and colour, and the evident reddish patch on the elytral disc in both species. It is, however, easily recognized by the latter for the multisetose abdominal sternite VII (in *C.splendidula*, the abdominal sternite VII bears only one seta on each side in males, two in females), generally larger size, and for the different shape of aedeagus.

##### Description.

The original description provided by Heller (1921), here completed with some additional characters, is sufficient to distinguish this taxon.

*General features* as in Figs 32–33. Medium-sized species: TL = 9.5–10.0 mm; L = 10.0–10.5 mm.

*Colour*: Head, antennae, pronotum, underside and legs reddish yellow. Elytra dark metallic green, with trace of bluish; disc with an evident reddish patch, one-half to two-thirds as long the elytra length, widest in apical third of elytra, occupying the inner five or six intervals; reddish patch not reaching elytral base, narrowed to apex, only with the first interval reddish at apex.

*Lustre and microsculpture*: Moderately shiny; head and pronotum with obsolete microsculpture; elytra with faint but distinct microsculpture in isodiametric meshes.

*Head*: Smooth; genae short, moderately swollen; neck constriction evident.

*Pronotum*: Wider than head, cordate, markedly transverse (ratio PW/PL = 1.17–1.23). Lateral margins widened and arcuate in front, reflexed and sinuate in the basal third; lateral expansions wide; posterior angles obtuse; disc with shallow transverse wrinkles. In one examined specimen, exceptionally the left side bears two antero-lateral setae.

*Elytra*: Moderately elongate (ratio EL/EW = 1.62–1.65), depressed, with basal border complete, extended to the parascutellar stria; striae deep, finely punctate; intervals convex at base, flattened on disc; intervals 7–8, slightly tumid at apex; apical margin obliquely truncate, with outer apical angles slightly thickened at the outer apical angles, which are obtusely rounded.

*Ventral side*: Abdominal sternites with sparse, short but evident pubescence. Abdominal sternite VII with two setae on each side in males, four on each side in females.

*Male genitalia*: As in Figs 34–35, median lobe of aedeagus slightly bent, its distal-middle part moderately dilated in dorsal view; dorsal and ventral margins slightly curved and undulate in lateral view; apical orifice pleuropic left; apical lamina flat, rounded at apex, slightly thickened in lateral aspect. Endophallus with two chitinized copulatory pieces, located in the apical half near the left lateral margin; the dorsal one larger in size, longish, markedly bent and acuminate at apex; the ventral one small, spine-like. Left paramere depressed on the dorsal side; right paramere not curved at apex.

*Female genitalia*: Not examined.

##### Geographical distribution and habitat.

Only known from the Mindanao Island in the Philippines (Map 2) from a very few specimens. Probably found in tropical forests.

##### Remarks.

We examined five other specimens from Philippines in the collection of BMNH, identified as “*discoidalis*”, which actually belong to *C.splendidula* (Fabricius).

#### [6] Calleida luzonensis

Taxon classificationAnimaliaColeopteraCarabidae

Casale & Shi sp. n.

http://zoobank.org/0AB89DFC-F6A5-49C7-A45D-219EA1AAEC0A

Figs 36–42 , Map 2

##### Type locality.

Philippines, Luzon, Nagtipunan (16.22N, 121.60E).

##### Type materials.

**Figures 36–42.**
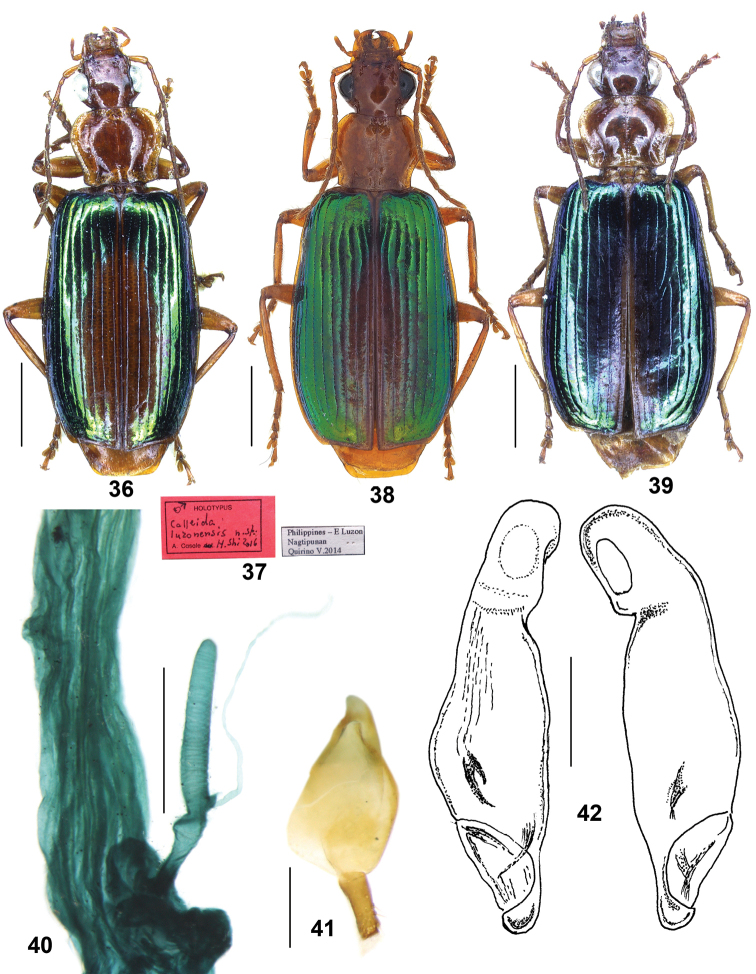
*Calleidaluzonensis* sp. n. **36** Habitus of holotype (male, Luzon, CCA) **37** Labels of holotype **38** Habitus (female paratype, Luzon, IZAS) **39** Habitus (female paratype, Luzon, CCA) **40** Female reproductive tract (paratype, Luzon, IZAS) **41** Gonocoxa (paratype, Luzon, IZAS) **42** Median lobe of aedeagus, dorsal and ventral views (holotype). Scale bars: 2.0 mm (**36, 38, 39**), 0.5 mm (**40, 42**), 0.2 mm (**41**).

**Holotype**: male, “Philippines-E Luzon Nagtipunan, Quirino V-2014” (CCA, Figs 36, 37). **Paratypes**: 2 females, same data as holotype, but “July 2014” and “August 2014” (CCA, CRS). 1 female, “Filippine VIII.2014 Dindin, Isabela, eastern Luzon, racc. loc. leg.” (CDG). 1 male, “Eastern Luzon, Sierra Madre, Madela, Tapsoy, Quirino”, “June 2016” (BMNH). 1 male, “Filippine: VIII.2014 Sierra Madre, Tapsoy, Quirino, Eastern Luzon, Ismael leg.” (CCA). 1 female, “Filippine: VIII.014 Nagtipunan, Quirino, Eastern Luzon, Ismael leg.” (CDG). 1 male, “Filippine: Tapsoy, Quirino, Eastern Luzon VIII.2014 (local collectors)” (CDG). 2 females, “Philippine: Luzon, Quirino, Maddela, Disimongal, Sierra Madre. Mar. 2016, local collector” (IZAS).

##### Specific epithet.

The new species is named after its type locality: Luzon Island, Philippines.

##### Diagnosis.

*C.luzonensis* sp. n. is distinct amongst all Asiatic *Calleida* species but close to *C.discoidalis* for: (1) elytra with evident reddish patch on the inner three to five intervals, markedly contrasting with the lateral metallic green intervals; (2) head, antennae, pronotum, legs and underside uniformly yellow reddish; (3) terminal ventrite with two setae on each side in males, three or more setae in females. The new species is closest to *C.discoidalis* from Mindanao Island, both having very similar external features. Comparing with *C.discoidalis*, *C.luzonensis* sp. n. has the following differences: (1) elytral discal reddish patch usually more reduced, narrower and more prolonged; (2) elytra metallic region generally lighter and more vivid; (3) body size generally a little larger (L = 10.5–11.0mm). The following differences in the median lobe of aedeagus support these two species as distinct: (1) in *C.luzonensis* sp. n. the median lobe smaller in size, shorter and stouter, length about 1.9 mm; in *C.discoidalis* median lobe length about 2.6 mm; (2) in *C.luzonensis* sp. n. apical lamina wider and less developed, length 0.6 time as the basal width, apex not thickened in lateral aspect, versus in *C.discoidalis*, apical lamina longer and more developed, length 0.7 times the basal width, apex a little thickened in lateral aspect; (3) left margin markedly prominent near middle in dorsal aspect in *C.luzonensis* sp. n., and evenly curved in *C.discoidalis*.

As with *C.discoidalis*, *C.luzonensis* might be confused with *C.splendidula*, a sympatric species in the Luzon Island (Philippines) based on their similar habitus and colour, and the evident reddish patch on the elytral disc in both species. Moreover, in one female paratype from Dindin, Isabela (Fig. 39), the reddish patch on the elytral disc is extended from the base to apex only on the sutural interval, and extended on the second and third intervals only near the apex. Therefore, the elytra appear almost completely metallic green and particularly similar to those of *C.splendidula* “var.” *unicolor* Jedlička, which is widely distributed in Philippines.

The new species is, however, easily recognized from the latter by the larger size, the multisetose abdominal sternite VII (in the species belonging to *C.splendidula* group, abdominal sternite VII bears only one seta on each side in males, two in females), and for the different aedeagal shape.

##### Description.

*General features* as in Figs 36, 38, 39. Medium-sized species: TL = 10.0–10.5 mm; L = 10.5–11.0 mm.

*Colour*: Head, antennae, pronotum, underside and legs bright reddish yellow. Elytra bright metallic green, disc with an evident reddish patch, generally one-half to two-thirds as long as the elytra length, widest in apical third of elytra, occupying the inner three to five intervals; reddish patch not reaching elytral base, narrowed to apex, only with the first interval reddish at apex. In one examined specimen (Fig. 39), the reddish patch is rather reduced, extended only to the second and third intervals, thus the elytra are almost completely metallic green.

*Lustre and microsculpture*: Dorsal surface moderately shiny and polished; head and pronotum with vanished microsculpture; elytra with faint but distinct microsculpture in isodiametric meshes.

*Head*: Slightly convex, almost impunctate; supraorbital furrows shallow, vanished a little behind the anterior edge of eyes; temporae moderately swollen, gradually narrowed towards the neck; genae shorter than half the length of eyes; antennae reaching the basal fifth of elytra; terminal labial palpomere strongly securiform with truncate apex in males, also dilated much less so in females.

*Pronotum*: Wider than head, transverse-cordate (ratio PW/PL = 1.19–1.24), depressed on disc. Lateral margins widened and arcuate in front, reflexed and sinuate in the basal third; lateral expansions wide; basal foveae wide, very deep; posterior angles obtuse; disc with shallow transverse wrinkles.

*Elytra*: Moderately elongate (ratio EL/EW = 1.52–1.65), slightly widened in the posterior third, depressed; basal border complete, extended to the parascutellar stria; striae punctate; intervals subconvex at base, flattened on disc, sparsely punctate; the eighth intervals slightly tumid near apex; apical margin obliquely truncate, weakly concave, with margin slightly thickened at the outer apical angle, which is obtusely rounded.

*Ventral side*: Abdominal sternites with sparse and short pubescence. Abdominal sternite VII with two setae on each side in males, three to five setae on each side in females.

*Male genitalia*: As in Fig. 42, median lobe of aedeagus with general features as in *C.discoidalis*, but smaller in size, L = 1.9 mm, more widened and stouter; apical orifice pleuropic left; left margin prominent near middle in dorsal aspect; apical lamina less developed, rounded distally, length about three-fifths as long as the basal width in dorsal aspect; apex not thickened in lateral aspect. Endophallus with two chitinized, small sized, spine-like copulatory pieces, located in the middle area near the left lateral margin, close to each other at base. Left paramere depressed on the dorsal side; right paramere not curved at apex.

*Female genitalia* (reproductive tract Fig. 40 and gonocoxa Fig. 41): Spermatheca digitiform, about two times length of pedicel, surface faintly whorled, with a very small basal projection; spermathecal pedicel straight, apex markedly swollen; spermathecal gland duct laterally inserted on the basal projection, about same length as spermatheca; glandular area not incrassate, about half length as gland duct, base with small protuberant atrium. Gonocoxite II sub-rectangular, 2.5 times long as the basal width, a little narrowed to apex, apex wildly truncate, not oblique; setose from the middle to apex on both inner and outer margins; outer margin a little curved; apex with membranous extension.

##### Geographical distribution and habitat.

Only known from eastern Luzon Island (Quirino and Isabela provinces) in the Philippines (Map 2). Probably found in tropical forests by local collectors.

## Phylogeny and character evolution

As a conclusion of the preliminary character analysis (see remarks under each species and species groups in text and our previous contribution: Casale and Shi 2018), we suggested that the female reproductive tract has important value in infrageneric taxonomy of *Calleida*. Moreover, in some species groups, the *C.splendidula* group in particular, the male genitalia are very homogeneous and make species delimitation difficult, but detailed studies of the female genitalia, especially the spermathecal characters, are expected to facilitate a solution in future studies. To interpret the evolution of female reproductive tract characters better, and to reveal the position of each species in the non-monophyletic *C.discoidalis* group, a very preliminary attempt of phylogenetic analysis was conducted.

### Characters and matrix

A total of 24 characters were selected, which included nine female genital characters, four male genital characters, four sexual dimorphic characters, and seven external characters. Two of the characters were multistate, whereas the others were binary. Although all characters were unordered in the phylogenetic analysis (without demonstration of character polarities), the supposed plesiomorphic states were coded “0”. A total of 18 species containing representatives of all nine species groups (defined in Casale and Shi 2018) of Asiatic *Calleida* was included in the phylogenetic analysis, but two of them lacked female or male genitalia characters respectively. The Australia-Asiatic genus *Anomotarus* was selected as out-group. The information on the matrix is found in Table 1. The character coding is shown below:

1 Spermathecal basal projection: (0) absent, (1) present.

2 Spermatheca basal sclerotized plate (*annulus receptaculi*): (0) absent, (1) present.

3 Whorl on spermatheca: (0) strong, (1) absent or very faint.

4 Apical protuberance of spermathecal pedicel: (0) absent, (1) present.

5 Spermathecal pedicel shape: (0) near straight, (1) strongly curved or curled.

6 Spermathecal pedicel length: (0) less than or subequal to spermatheca, (1) longer than spermatheca.

7 Spermathecal gland duct insertion: (0) ventrally, (1) laterally.

8 Spermathecal glandular area: (0) not or weakly incrassate, (1) distinctly inflated.

9 Gonocoxite II apex: (0) subulate, (1) oblique truncate.

10 Ventral lobe on aedeagus: (0) absent, (1) present.

11 Male aedeagus venter: (0) plain or lobed, (1) ridged or markedly concaved.

12 Primary (ventral) copulatory piece of endophallus: (0) shorter than half length of median lobe, (1) longer than length of median lobe, flagellum-like, (2) absent or very weakly defined.

13 Secondary (dorsal) copulatory piece of endophallus: (0) well chitinized, (1) absent or very weakly defined.

14 Apex of abdominal sternite VII in males: (0) straight, (1) notched.

15 Setae on each side of male abdominal sternite VII: (0) one seta, (1) normally two, exceptionally three setae, (2) three or more setae.

16 Setae on each side of female abdominal sternite VII: (0) two setae, (1) three or more setae.

17 Female terminal labial palpomere: (0) same as in males, securiform, (1) less dilated than in males.

18 Antennomeres I–III except primary setae: (0) glabrous, (1) with accessory setae.

19 Pronotum anterior angles: (0) glabrous, (1) setose.

20 Elytra: (0) uniformly metallic, (1) disc with reddish patch.

21 Elytral apical margin: (0) straight or weakly concaved, (1) strongly concaved.

22 Elytral outer apical angles: (0) rounded or obtuse, (1) sharply angulate.

23 Elytral margins on apical outer angles: (0) more or less thickened, (1) not thickened.

24 Ratio elytra length/width: (0) between 1.5 to 1.75, (1) greater than 1.8.

**Table 1.** Characters matrix for Asiatic *Calleida* and the out-group *Anomotarusstigmula*. “?” = missing data.

Taxa / Character	000000000111111111122222
	123456789012345678901234
*Anomotarusstigmula* (Chaudoir)	000000011002000010000010
*C.gressittiana* Casale & Shi	?????????001110011100010
*C.puncticollis* Shi & Casale	000000000002102100000010
*C.excelsa* Bates	000000111000010011110011
*C.jelineki* Casale & Shi	000000111000010010100011
*C.corporaali* Andrewes	011000101100110010001100
*C.viet* Casale & Shi	011000101100110010001100
*C.borneensis* Shi & Casale	101111101000010010001000
*C.doriae* Bates	101100100010010010000000
*C.lepida* Redtenbacher	101101100010010010000000
*C.sultana* Bates	101101100010010010100000
*C.cf.splendidula* (Fabricius)	101011101000010010010000
*C.tenuis* Andrewes	101011101000010010000000
*C.onoha* Bates	101001101000010010000000
*C.luzonensis* sp. n.	100000101000011110010000
*C.cochinchinae* sp. n.	0000001?1??????100010011
*C.piligera* sp. n.	011000100000012110000000
*C.fukiensis* Jedlička	100000101000011110000000
*C.yunnanensis* sp. n.	100000101100011110010000

Most of the materials used in the phylogenetic analysis were cited in the present and our previous contribution (Casale and Shi 2018). Localities for examined materials of species not cited previously are shown below:

*Anomotarusstigmula* (Chaudoir): India (Andhra Pradesh)

*Calleidalepida* Redtenbacher: China (Jiangxi)

*Calleidasultana* Bates: China (Yunnan)

*Calleida* cf. *splendidula* (Fabricius): China (Guangxi)

*Calleidatenuis* Andrewes: Malaysia (Sabah)

*Calleidaonoha* Bates: Japan (Okinawa)

### Phylogenetic analysis

The phylogeny reconstruction was performed using WIN-PAUP* Version 4.0b10 with the following parameters: Optimality criterion = parsimony; all characters were unordered; starting tree(s) was obtained via stepwise addition; addition sequence: random; number of replicates = 1000; number of trees held at each step during stepwise addition = 10; branch-swapping algorithm: TBR; steepest descent option not in effect; initial ‘MaxTrees’ setting = 100; ‘MulTrees’ option in effect; topological constraints not enforced; trees unrooted; bootstrap method with heuristic search; and number of bootstrap replicates = 1000. Branches with bootstrap values greater than 50% were maintained.

**Figure 43.**
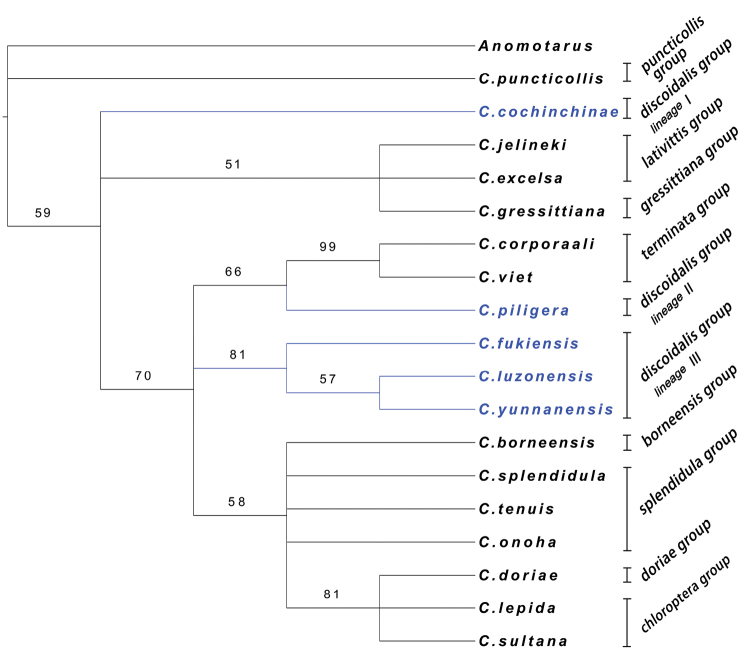
Cladogram of Asiatic *Calleida* species relationships obtained by the SW method with bootstrap values greater than 50%. Species group membership is shown in the right side. The polyphyletic *C.discoidalis* species group is in blue.

Both the equal weighting (EW) method and the successive weighting (SW) method were used in the phylogeny reconstruction. But, because of a limited number of characters, several branches in the cladogram with EW method were not expanded. So, only the cladogram generated with the SW analyses is presented in Fig. 43. In the SW analysis, 66 most parsimonious trees were obtained. The length and indices for each most parsimonious tree were as follow: Tree length = 25.6–26.4; consistency index (CI) = 0.682–0.704; retention index (RI) = 0.812–0.830; and rescaled consistency index (RC) = 0.554–0.584.

## Results

In the cladogram generated with the SW method, the monophyly for each species group was corroborated with the exception of the *C.discoidalis* group which was posited as a polyphyletic group (blue color branches in the cladogram). Several relationships among species groups were suggested with relatively high reliabilities.

In our previous contribution (Casale and Shi 2018), two rather isolated lineages in the Asiatic *Calleida* were proposed mainly on their special male endophallus characters. One of them, the *C.puncticollis* species group, formed the earliest branch of Asiatic *Calleida* in the cladogram. In contrast, another primary lineage, the *C.gressittiana* group, did not form an isolated clade in the cladogram. Instead, it was grouped in the *C.lativittis* group, but because *C.gressittiana* is the only species with female genitalia unknown, this relationship is questionable.

The polyphyletic *C.discoidalis* group was composed of three isolated lineages. **Lineage I** containing only one known species, *C.cochinchinae*, was suggested as a relatively early branch in the cladogram. A monophyletic or paraphyletic group of lineage I + *C.lativittis* group was presumed, supported by the similarities on female reproductive tract shape (type II) and elongate elytra (character 24). **Lineage II** containing only one known species, *C.piligera*, was suggested as the sister group of the *C.terminata* group by a moderately high bootstrap value (= 66). Such relationship was also well founded by the presence of the basal plate on female reproductive tract (type IV). **Lineage III** containing four known species (three species selected in the phylogenetic analysis, which belong to the *C.discoidalis* group). The monophyly of lineage III was well supported (= 81) and can be also inferred by the similar female reproductive tracts (type IV).

Five species groups and two lineages of the *C.discoidalis* group formed a monophyletic clade (= 70), containing more than 80% described species of Asiatic *Calleida*. Under this clade, the *C.splendidula* group, *C.borneensis* group, *C.doriae* group, and *C.chloroptera* group are closer to each other than to the other branches. The closer relationships among them were also supported by their similar female reproductive tracts (type V).

The monophyly of these four groups and their relationships were unresolved in the cladogram, but it was suggested that the *C.doriae* group + *C.chloroptera* group are monophyletic (= 81), and *C.borneensis* is close to *C.splendidula* group.

### Character evolution for female reproductive tract

**Figure 44.**
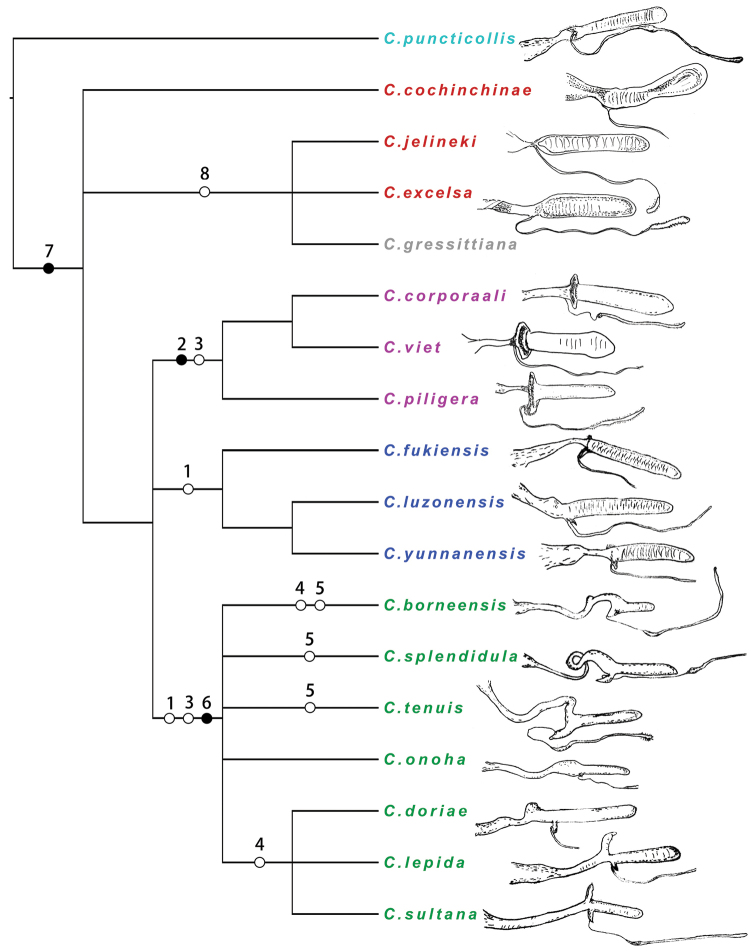
Possible character evolutions for female reproductive tracts in Asiatic *Calleida* species. Solid spots represent apomorphies; empty circles represent homoplasy. Five types of spermathecae are shown in different colors on taxa: type I (cyan), type II (red), type III (purple), type IV (blue), type V (green).

The character evolution analysis was based on the cladogram obtained with the SW analysis. In this cladogram, only the eight characters (1–8) of female reproductive tract were analyzed. Character evolution was marked on the branches. For homoplastic transformations, the maximum parsimonious assumption was accepted with parallelisms priority to reversals. Spermathecae for all available species contained in the phylogenetic analysis were illustrated in Fig. 44.

Three of the eight female reproductive tract characters were considered having apomorphic conditions (solid spots in Fig. 44), with spermathecal gland duct insertion (char. 7) transformed from ventrally to laterally, which supported the *C.puncticollis* group as the earliest branch of Asiatic *Calleida*. The presence of a well sclerotized basal plate (char. 2) supported the monophyly of the *C.terminata* group + *C.discoidalis* group lineage II, both with well defined type III spermathecae. The elongate spermathecal pedicel (char. 6) supported a monophyletic clade of four species groups (*C.splendidula*, *C.borneensis*, *C.doriae*, and *C.chloroptera* groups) with spermatheca type V.

The remaining five characters had homoplastic or ambiguous transformations (empty circles in Fig. 44). Two of them (char. 1 and char. 3) were considered important, supporting three monophyletic clades (corresponding to female reproductive tract type III, type IV, and type V). But, the apomorphic transformation assumptions of these two characters were conflicted on solving relationships among these three clades. Two other characters (char. 4 and char. 5) only transformed inside the monophyletic clade of *C.splendidula* group + *C.borneensis* group + *C.doriae* group + *C.chloroptera* group, but they did not support a phylogenetic relationship except for the monophyly of *C.doriae* group + *C.chloroptera* group, which was supported by the presence of apical protuberance of spermathecal pedicel (char. 4).

### Female reproductive tract category

Five types of female reproductive tracts were recognized corresponding to five lineages in the cladogram (different colors in Fig. 44). Four of them were suggested as monophyletic, while the monophyly of the type II was neither supported nor objected by the cladogram.

**Type I** Spermatheca distinctly whorled; spermathecal gland duct ventrally inserted; spermathecal base and pedicel apex not modified. Type I was supposed to be a very special and isolated type in Asiatic *Calleida*, only represented by the *C.puncticollis* group. In this species, also the absence of copulatory piece in endophallus is unique.

**Type II** Similar to type I, but spermathecal gland duct laterally inserted. Present in the *C.lativittis* group and lineage I of the *C.discoidalis* group. The type II also showed several primary characters, but can be separate from type I by the different position of spermathecal gland duct insertion.

**Type III** Spermatheca not or very faintly whorled; spermatheca with a well sclerotized basal plate (*annulus receptaculi*); spermathecal pedicel narrow and short. Present in the monophyletic clade of *C.terminata* group + *C.discoidalis* group lineage II. Type III is well recognized for its highly modified *annulus receptaculi*. The modified spermathecal base and the not or very faintly whorled spermatheca suggest a relationship to the type V.

**Type IV** Spermatheca distinctly whorled, with a distinct basal projection; spermathecal pedicel short and partly expanded; atrium very weakly defined. Present in lineage III of the *C.discoidalis* group.

**Type V** Present in the monophyletic clade of *C.splendidula* group + *C.borneensis* group + *C.doriae* group + *C.chloroptera* group, containing more than half of described Asiatic *Calleida* species. Type V is a highly modified and diverse type, recognized by the not or very faintly whorled spermatheca, strongly elongate spermathecal pedicel, and the presence of spermathecal basal projection. The elongate spermathecal pedicel is curved or curled, and/or has an apical protuberance in some taxa. The variable spermathecal pedicel in type V could be important in species definition of the *C.splendidula* group.

## Conclusion

The phylogeny reconstruction partly supported the monophyly of most species groups, but the *C.discoidalis* group was proved to be polyphyletic with three isolated lineages. Some relationships amongst species groups were solved, but many branches had relatively low bootstrap value, while some others were unexpanded. Because of the above insufficiency, we did not tend to revise the definition of species groups on the phylogeny results, and just regard it as a very preliminary attempt to reveal species relationships.

The results of character evolution analysis showed that the female reproductive tract has very important taxonomic value in *Calleida*. Five distinct types of female reproductive tracts were recognized, corresponding to four monophyletic and one paraphyletic branches in the cladogram. Future studies are expected to solve character transformation polarity and better evaluate taxonomic value, when more materials will be examined, such as Afrotropical and Neotropical *Calleida* species, and allied genera of Calleidina such as the African genus *Lipostratia* Chaudoir and the Australian genus *Demetrida* White.

## Supplementary Material

XML Treatment for Calleida fukiensis

XML Treatment for Calleida piligera

XML Treatment for Calleida cochinchinae

XML Treatment for Calleida yunnanensis

XML Treatment for Calleida discoidalis

XML Treatment for Calleida luzonensis

## References

[B1] Casale A (1998) Phylogeny and biogeography of Calleidina (Coleoptera: Carabidae: Lebiini): a preliminary survey. In: Ball GE Casale A Vigna Taglianti A (Eds) Phylogeny and Classification of Caraboidea.Proceedings of a Symposium. 28 August, 1996, Florence, Italy. XX International Congress of Entomology. Atti Museo regionale di Scienze naturali, Torino, 381–428.

[B2] Casale A Shi HL (2018) Revision of the Oriental species of *Calleida* Latreille (sensu lato). Part 1: introduction, groups of species, and species of six species groups (Coleoptera: Carabidae: Lebiini). Zootaxa 4442(1): 1–42. 10.11646/zootaxa.4442.1.1 30313981

[B3] Heller KM (1921) New Philippine Coleoptera. The Philippine Journal of Science 19: 523–627. 10.5962/bhl.part.1235

[B4] Kabak I (2017) Lebiini: Calleidina. In: Löbl I Löbl D (Eds) Catalogue of Palaearctic Coleoptera.Vol. 1. Archostemata – Myxophaga – Adephaga. Revised and Updated Edition. Brill NV, Leiden, 580–582.

[B5] Jedlička A (1953) Neue Carabiden aus der chinesischen Provinz Fukien. Entomologische Blätter, Krefeld 49: 141–147.

[B6] Jedlička A (1963) Monographie der Truncatipennen aus Ostasien, Lebiinae-Odacanthinae- Brachyninae (Coleptera, Carabidae). Entomologische Abhandlungen und Berichte aus dem Staatlichen Museum fuer Tierkunde in Dresden 28: 269–579.

[B7] Kirschenhofer E (1986) Neue Arten truncatipenner Carabidae der palaearktischen und orientalischen Region unter besonderer Berucksichtigung der Aufsammlungen Eigin Suensons in Ostasien (Coleoptera, Carabidae). Entomofauna 7(23): 317–348.

[B8] Lorenz W (2005) Systematic List of Extant Ground Beetles of the World (InsectaColeopteraGeadphaga: Trachypachidae and Carabidae Incl. Paussinae, Cicindelinae, Rhysodinae), second edition. Wolfgang Lorenz, Tutzing, Germany, 530 pp.

